# The role of cementoblasts in root resorption induced by orthodontic treatment: from mechanosensation to repair potential

**DOI:** 10.3389/fcell.2026.1953825

**Published:** 2026-09-24

**Authors:** Yue Feng, Yanling Xie, Xing Hu, Zhiqiao Qiu, Shuixue Mo

**Affiliations:** 1 College & Hospital of Stomatology, Guangxi Medical University, Nanning, China; 2 Department of Orthodontics, College & Hospital of Stomatology, Guangxi Medical University, Nanning, China; 3 Guangxi Key Laboratory of Oral and Maxillofacial Rehabilitation and Reconstruction, Nanning, China

**Keywords:** cementoblast, cementum regeneration, mineralization, orthodontic tooth movement, orthodontically induced inflammatory root resorption

## Abstract

While alveolar bone has high remodeling activity under physiological conditions, cementum has a strong antiresorptive capacity. Cementoblasts are the primary functional and mechanosensitive cells on the cementum surface that maintain the structural homeostasis of the tooth root. However, excessive or sustained orthodontic force applied during treatment often induces irreversible orthodontically induced inflammatory root resorption (OIIRR), characterized by impaired cementoblast function and defective root surface repair. Although the precise molecular mechanisms underlying cementoblast dysfunction and the progression of root resorption under orthodontic force remain incompletely understood, posing major challenges for the prevention and management of OIIRR, orthodontic force-induced programmed cell death and secretory dysregulation in cementoblasts are known to hinder the self-repair of resorption lacunae, thereby exacerbating root tissue destruction. Consequently, intervention strategies—including physical therapy, pharmacological therapy, and biomaterial-based approaches—targeting the biological properties of cementoblasts are being developed to reactivate their differentiation and mineralization potential and promote functional repair of the root surface. This review provides a comprehensive overview of the biological role of cementoblasts in orthodontic root resorption and repair, establishing a theoretical basis and providing new insights for the development of cementoblast-targeted strategies to prevent and treat OIIRR.

## 1 Introduction

Malocclusion is a prevalent oral condition that can impair dental health, facial aesthetics, and patient psychological wellbeing. Orthodontic treatment is the primary approach for correcting malocclusion. However, several complications can arise during treatment, including orthodontically induced inflammatory root resorption (OIIRR), enamel demineralization, and periodontitis. OIIRR—the progressive loss of hard tissue in the apical root region caused by mechanical forces—is the most common of these complications, affecting approximately 80%–90% of orthodontic patients (Neves et al., 2022; Zhou et al., 2023). Because resorption in the apical third of the root is generally considered irreversible (Heboyan et al., 2022), severe OIIRR can result in marked root shortening, tooth mobility, impaired masticatory function, and tooth loss. This condition not only compromises oral health but also notably increases the complexity and financial burden of subsequent dental restoration. Therefore, understanding the mechanisms underlying root resorption is essential for developing effective preventive and therapeutic strategies.

From a biological perspective, orthodontic tooth movement (OTM) relies on continuous alveolar bone remodeling. In contrast, cementum, which anchors the tooth, must remain relatively stable during this dynamic process to prevent root damage. This protective barrier function is primarily mediated by cementoblasts on the cementum surface. These cells secrete the cementum matrix and regulate its mineralization, thereby preserving cementum integrity and ensuring the functional attachment of periodontal ligament fibers (Wang H. et al., 2020; Wang H. et al., 2023). Under physiological conditions or after minor injury, cementoblasts sense mechanical stimulation and rapidly activate self-repair mechanisms to fill small resorption lacunae. However, under excessive or continuous orthodontic force, cementoblasts exhibit reduced secretory and mineralization capacities or even undergo apoptosis. This triggers osteoclast activation and recruitment, leading to the removal of the protective barrier on the root surface and ultimately causing root resorption (Diercke et al., 2014; Wang et al., 2025). Additionally, cementoblast function is tightly regulated by surrounding cells, including macrophages and periodontal ligament cells (Huang et al., 2024; Li et al., 2024). This suggests that cementoblasts and surrounding cells within the periodontal microenvironment collectively regulate root resorption and repair under orthodontic force (Sawada et al., 2024).

Recent studies targeting cementoblasts have demonstrated that enhancing their differentiation and mineralization promotes cementum repair and mitigates OIIRR. For instance, low-intensity pulsed ultrasound (LIPUS) counteracts the compressive force-induced suppression of mineralization (Dalla-Bona et al., 2008; Inubushi et al., 2013; Zheng et al., 2025), while pharmacological agents such as parathyroid hormone (PTH) and resveratrol (Li et al., 2021a; Negm et al., 2025) have shown potential in protecting cementoblasts and attenuating root resorption (Li et al., 2021a; Negm et al., 2025). Additionally, biomaterial-based strategies, such as the use of bioactive peptide hydrogels, promote cell migration, ameliorate the immune microenvironment, and induce cementum mineralization through direct interaction with cementoblasts, establishing a framework for guiding the design of periodontal tissue-specific regenerative scaffolds.

Based on recent research, this review systematically summarizes the biological roles of cementoblasts in OIIRR, their underlying regulatory mechanisms, and potential intervention strategies, establishing a theoretical foundation and new insights for the prevention and treatment of OIIRR. To synthesize the relevant evidence, literature searches were conducted in PubMed and Web of Science using the following search terms and Boolean operators (“AND”, “OR”): (“cementoblast*” or “cementoblastic”), (“orthodontic*” or “tooth movement” or “orthodontic tooth movement” or “OTM”), and (“root resorption” or “orthodontically induced inflammatory root resorption” or “OIIRR” or “EARR”).

## 2 The mechanism underlying OIIRR pathophysiology

OIIRR is a complex process involving multiple cell types and crosstalk among signaling pathways. It progresses through three distinct stages of OTM: the initial movement phase, the lag phase, and the post-lag phase (Figure 1).

**FIGURE 1:**
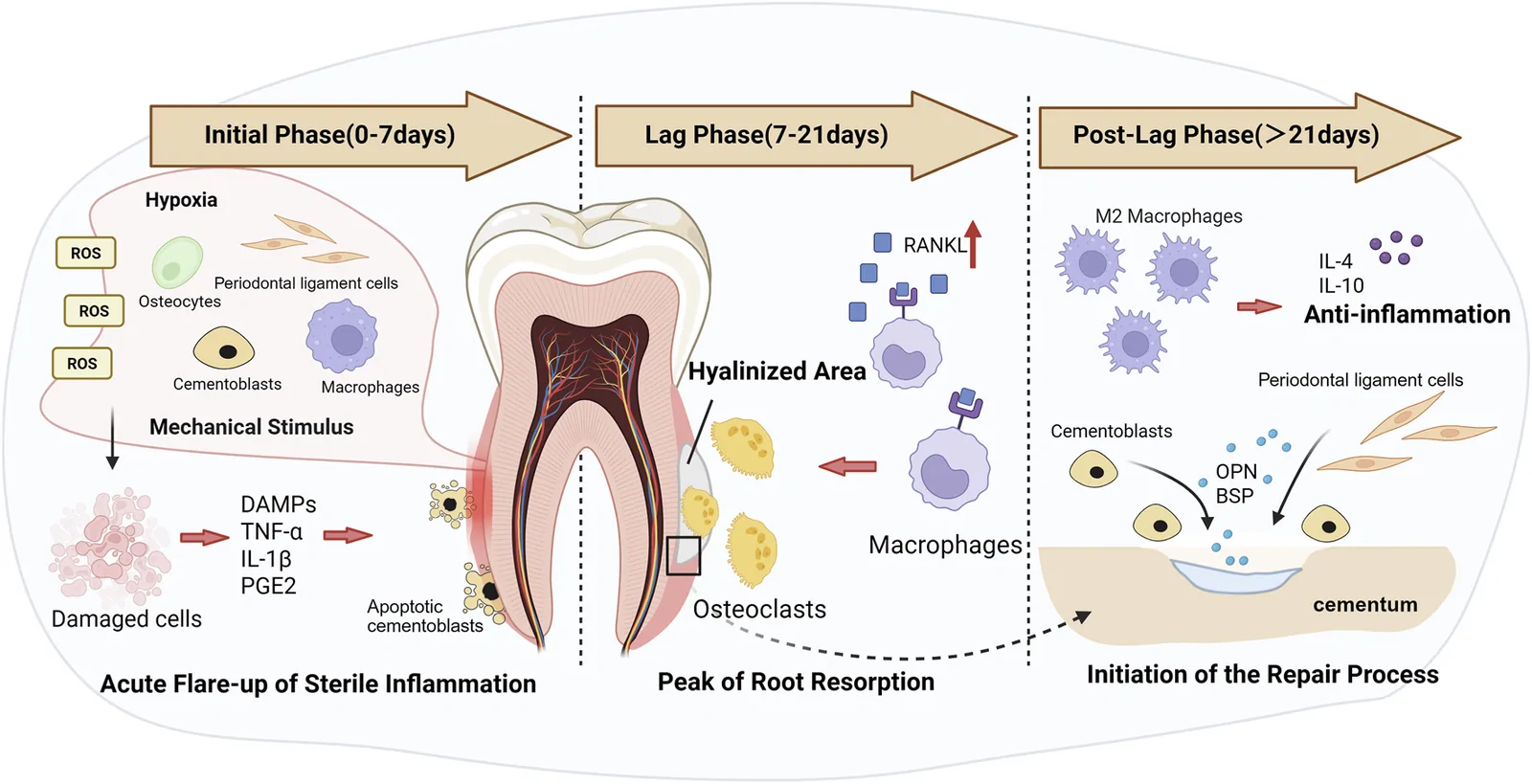
Schematic illustration of cellular and molecular events at distinct stages of orthodontically induced inflammatory root resorption (OIIRR): the initial phase (acute inflammation), the lag phase (peak of resorption), and the post-lag phase (repair initiation). Created in BioRender. yue, F. (2026) https://BioRender.com/665s5zh.

Within the first 7 days of orthodontic force application, teeth move rapidly within the periodontal space, accompanied by local hypoxia and reactive oxygen species accumulation on the compression side of the periodontal ligament (Steinberg et al., 2025). During this phase, periodontal ligament cells and macrophages undergo pyroptosis, whereas osteocytes undergo necroptosis. These cell death processes release substantial amounts of damage-associated molecular patterns and pro-inflammatory mediators—including tumor necrosis factor-alpha (TNF-α), interleukin-1 beta (IL-1β), and prostaglandin E2 (PGE2)—which recruit M1-type macrophages to the compression zone (Gong et al., 2020; Li Y. et al., 2021; Ohori et al., 2026). Growing evidence supports that cementoblasts are mechanosensitive, and that orthodontic force can impair their survival and mineralization capacity via the mechanosensitive ion channel Piezo1, thereby exposing the root surface and weakening the protective cementum barrier (Zhang et al., 2017; Huang et al., 2025; Wang et al., 2026). As blood vessels on the compression side become completely occluded, a sterile necrotic hyalinization zone forms locally, transitioning tooth movement into the lag phase. During this phase (approximately 7–21 days), phagocytic cells—including macrophages, osteoclast precursors, and multinucleated giant cells—are recruited to the necrotic area. Their removal of necrotic tissue inadvertently erodes the underlying root surface, creating cementum defects (Brudvik and Rygh, 1993; 1994). Simultaneously, elevated local levels of PGE2, IL-17, and IL-34 promote osteoclast differentiation and maturation by upregulating receptor activator of nuclear factor-kappa B ligand (RANKL), thereby maximizing the risk of root resorption (Park et al., 2017; Kitaura et al., 2025; Shen et al., 2026).

During the recovery (post-lag) phase, the removal of necrotic tissue is followed by an increase in M2 macrophage recruitment and the upregulation of anti-inflammatory cytokine expression (IL-4, IL-10), which together establish a reparative microenvironment (Iglesias-Linares and Hartsfield, 2017). Periodontal ligament fibroblasts secrete non-collagenous proteins such as osteopontin (OPN) and bone sialoprotein (BSP) that occupy the remaining collagen framework within resorption lacunae (Jager et al., 2008). The exposed extracellular matrix, comprising proteins such as fibronectin and OPN, then promotes the migration of cementoblast precursors to the root surface, where they secrete type I collagen and cementum-specific proteins such as cementum protein 1 (CEMP-1) and cementum attachment protein (CAP) under the regulation of RUNX2 and osterix (OSX). This process promotes the formation of an acellular cementum-like matrix and promotes hydroxyapatite deposition, ultimately generating cellular intrinsic fiber cementum and restoring the structure and function of cementum and periodontal supporting tissues (Li et al., 2026). However, when orthodontic forces are excessive or prolonged, osteoclast/odontoclast activity surpasses the rate of repair, and root resorption persists (Rody et al., 2019).

In summary, the structural integrity of the root depends on the dynamic coupling between cementoblast anabolism and osteoclast/odontoclast catabolism. Under orthodontic force, the destructive activity of osteoclasts/odontoclasts exceeds the upper limit of barrier protection and reparative compensation provided by the cementoblast layer, thereby driving pathological resorption of root tissue (Rostami et al., 2026). Therefore, elucidating the mechanisms underlying cementoblast damage and repair under orthodontic force—and developing targeted regenerative intervention strategies—holds key theoretical and clinical significance for reducing the incidence of orthodontically induced root resorption and improving the long-term safety of orthodontic treatment.

## 3 The role of cementoblasts and the mechanisms governing their responses in OIIRR

Cementoblasts are protein-synthesizing secretory cells primarily distributed along the cementum surface and cementoid layer. Their core role is to preserve functional periodontal ligament attachment and provide an antiresorptive barrier on the root surface by continuously secreting extracellular matrix, thereby maintaining the integrity of the cementum layer (Zhao et al., 2004; Yamaguchi and Fukasawa, 2021). Cementoblasts synthesize an organic matrix composed mainly of type I collagen (approximately 90%) and secrete non-collagenous enzymes and other proteins such as BSP, OPN, osteocalcin (OCN), and alkaline phosphatase (ALP) (Diercke et al., 2012b), which contribute to matrix organization and the regulation of mineralization (Arzate et al., 2015). These fundamental activities allow cementoblasts to sustain periodontal homeostasis under physiological conditions. However, under orthodontic force, normal cementoblast function is impaired. The activation of multiple cytokines and signaling pathways disrupts the integrity of this protective barrier and influences the onset and progression of OIIRR (Figure 2). In Table 1, we summarize the roles of cementoblasts and the mechanisms governing their responses in OIIRR—including programmed cell death, autophagy inhibition, interactions with the inflammatory microenvironment, and repair processes—along with their associated signaling pathways and cellular effects.

**FIGURE 2:**
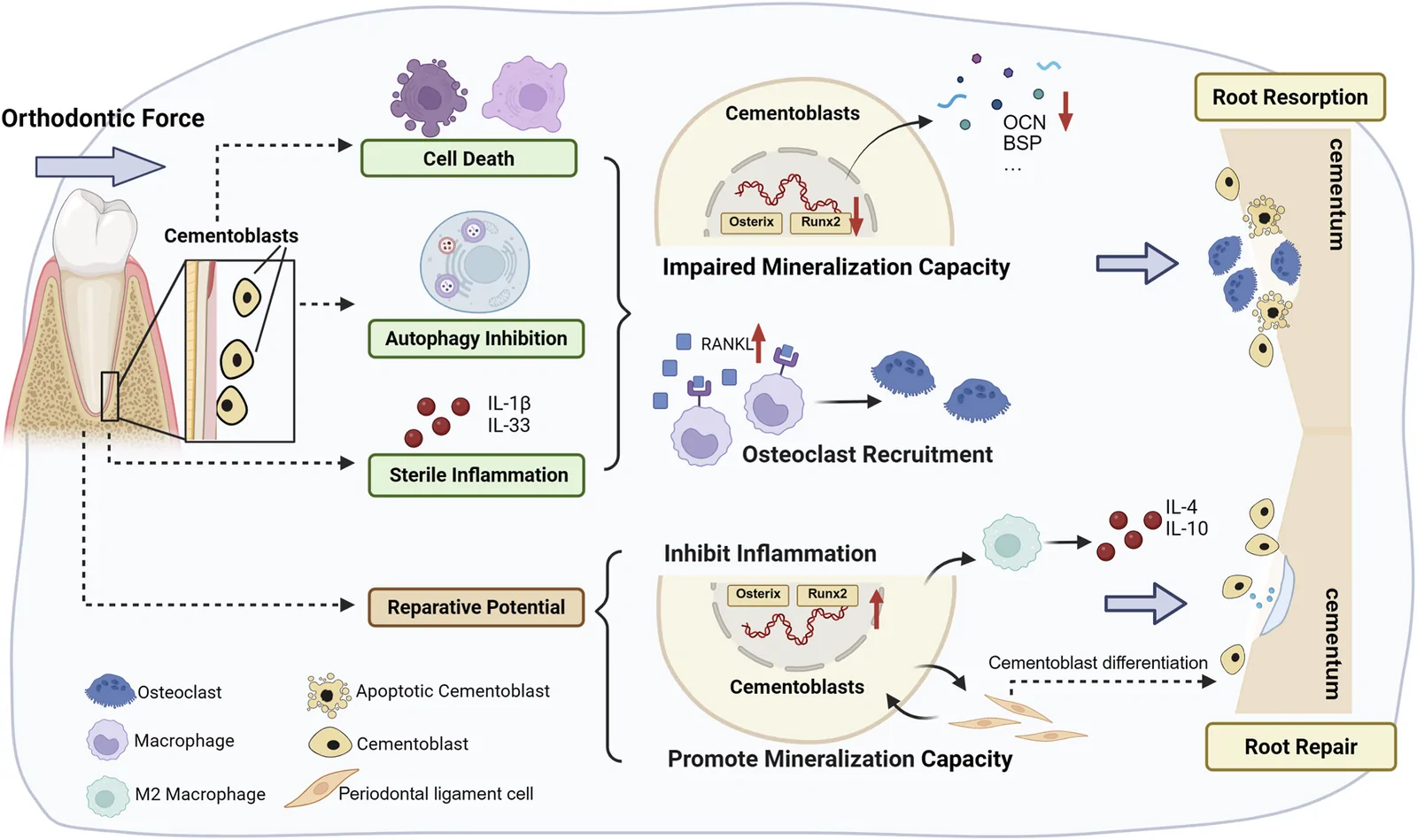
Schematic diagram of the role of cementoblasts and the mechanisms governing their responses in orthodontically induced inflammatory root resorption (OIIRR). Cementoblasts play key roles in OIIRR, promoting root resorption and regenerative repair. Created in BioRender. yue, F. (2026) https://BioRender.com/98zb5iu.

**TABLE 1:** The role of cementoblasts and the mechanisms governing their responses in orthodontically induced inflammatory root resorption (OIIRR).

Role	Experimental model	Growth factor/protein/pathway	Core cellular effects	Result	References
Programmed cell death	*In vitro* (human primary cementoblasts)	AXIN1/AXUD1, JNK	Promote apoptosis	Mechanical stress directly triggers cementoblast apoptosis	Diercke et al., 2014
	*In vitro* (OCCM-30)	ERK1/2	Promote (↑PGE2, COX2)	Leptin amplifies stress-induced mitochondrial apoptosis and inflammation, exacerbating resorption	Ruiz-Heiland et al., 2021
	*In vitro* (OCCM-30)	HIF-1/FOXO	Inhibitory (↓RUNX2, GPX4, ALP)	Compressive stress alters lncRNAs, inducing hypoxia/apoptosis and inhibiting osteogenesis	Liu et al., 2019
	*In vitro* (OCCM-30)/*in vivo* (mouse)	cGAS-STING/GPX4	Promotive (↑ROS, Fe^2+^, MDA); inhibitory (↓GPX4)	Compressive force induces ferroptosis, inhibits cementoblast mineralization	Wei et al., 2026
	*In vivo* (rat)	Caspase-8/3	Promote (↑Caspase-8/3, TRAP^+^ cell)	Heavy force induces apoptosis, which precedes osteoclast activation and root resorption	Minato et al., 2018
Autophagy inhibition	*In vitro* (OCCM-30)	LincRNA-p21	Inhibitory (↓LC3-II/I, BECN1, OSX, RUNX2; BSP, OCN)	Compressive force inhibits autophagy via lincRNA-p21, impairing mineralization	Liu H. et al., 2022
	*In vitro* (OCCM-30)/*in vivo* (mouse)	β-Catenin	Inhibitory (↓Postn, β-catenin, Wnt targets)	Autophagy maintains mineralization via Postn/β-catenin; autophagy inhibition worsens stress-induced resorption	Yang et al., 2023
	*In vitro* (OCCM-30)/*in vivo* (rat)	S1PR1	Promotive (↑ROS/mtROS) Inhibitory (↓Runx2, OSX, ALP)	Causing mitochondrial dysfunction and promoting resorption	Wang et al., 2025
Interaction between the inflammatory microenvironment and cementoblasts	*In vitro* (human cementoblasts)	IL-1β	Inhibitory (↓BSP, CEMP-1)	Synergy of IL-1β and compression suppresses cementoblast markers (BSP, CEMP-1), reducing repair capacity	Diercke et al., 2012b
	*In vitro* (human cementoblasts)	COX-2/PGE2	Promotive (↑RANKL, COX-2, RANKL/OPG)	IL-1β and compression synergistically upregulate RANKL expression via COX-2, inducing osteoclastogenesis and severe resorption	Diercke et al., 2012a
	*In vitro* (OCCM-30)	MiR-155-3p, Wnt/β-catenin	Inhibitory (↓BSP, Spp1, Bglap, OSX)	TNF-α inhibits differentiation/mineralization via miR-155-3p/*Kctd1* and mediates inflammatory tissue destruction	Wang et al., 2017
	*In vitro* (OCCM-30)/*in vivo* (mouse)	IL1-R1, IL1-R2	Inhibitory (↓BSP, CEMP-1)	IL-1β-stimulated cementoblasts can support osteoclastogenesis	Huynh et al., 2017
​	*In vitro* (OCCM-30)/*in vivo* (mouse)	MiR-325-3p, RUNX2	Inhibitory (↓RUNX2, OSX; ALP)	IL-1β inhibits differentiation/mineralization by upregulating miR-325-3p, which targets RUNX2	Wang Y. et al., 2020
	*In vitro* (OCCM-30)/*in vivo* (mouse)	Chemerin/ChemR23	Promotive (↑TNF-α, IL-6) Inhibitory (↓RUNX2, OSX; OPG, BSP, OCN)	Chemerin/ChemR23 induces inflammatory cytokines via MAPK/PI3K-AKT, inhibiting osteogenesis and promoting osteoclastogenesis, worsening resorption	Ye et al., 2021
	*In vitro* (OCCM-30)/*in vivo* (mouse)	FTO, RUNX2	Inhibitory (↓RUNX2, FTO)	TNF-α inhibits differentiation via m6A modification, which drives pathological cementogenesis	Sun et al., 2022
	*In vitro* (OCCM-30)/*in vivo* (mouse)	IL-33/ST2	Inhibitory (↓RUNX2; OSX, BSP, OPN, OPG/RANKL)	Force-induced IL-33 has anti-reparative effects, exacerbating resorption by inhibiting differentiation and promoting osteoclastogenesis	Dong et al., 2020
Repair	*In vitro* (RAW264.7)	Histone H3 hyperacetylation	Promotive (↑ARG1, IL-10, H3 acetylation)	Orthodontic pressure promotes macrophage M2 polarization via H3 acetylation	Wang H. et al., 2023
	*In vitro* (OCCM-30)	PI3K/AKT	Promotive (↑COL1, RUNX2, OSX; CAP, OCN)	HPDLSC exosomes enhance cementoblast activity	Li et al., 2024
	*In vitro* (OCCM-30)	Stem cell paracrine effects	Promotive (↑ALP, MMP-9, MMP-13)	Dental mesenchymal stem cells have direct reparative effects on cementoblasts	Li Wenwen et al., 2025
	*In vitro* (OCCM-30)	PTH1R and ERK1/2	Promotive (↑PTHrP, PTH1R, RUNX2, OSX; COL1, OPN)	PTH1R/ERK1/2 is essential for stretch-induced cementoblast differentiation	Wang H. et al., 2023
	*In vitro* (OCCM-30)	HIF-1α regulation of PD-L1	Inhibitory (↓PD-L1)	Cementoblasts have immunomodulatory roles in hypoxia; HIF-1α/PD-L1 potentially mediate immunosuppression and resorption	Yong et al., 2022b
	*In vivo* (mouse)	Wnt/β-catenin	Promotive (↑BSP, ALP)	Cementoblasts promote PDL cell differentiation into cementoblasts via juxtacrine signaling, initiating repair	Sawada et al., 2024
	*In vitro* (OCCM-30)/*in vivo* (mouse)	M2 macrophage-derived exosomes	Promotive (↑OSX, RUNX2)	Macrophage polarization bidirectionally regulates cementoblast mineralization via exosomes; alleviates root resorption *in vivo*	Zhao et al., 2022
​	*In vitro* (OCCM-30)/*in vivo* (mouse)	Exosomal Let-7f-5p	Promotive (↑PGC-1α)	Engineered M2 exosomes modulate mitochondrial function, promoting cementum regeneration	Huang et al., 2024
	*In vitro* (OCCM-30)/*in vivo* (mouse)	Comp-EVs modulate macrophage behavior	Comp-EVs promote macrophage M2 polarization and efferocytosis	Compressed cementoblasts modulate macrophage-mediated repair through EV release, providing insights into antiresorptive mechanisms	Yang et al., 2024
	*In vitro* (OCCM-30)/*in vivo* (mouse)	β-Catenin	Autophagy activators restore mineralization and mitigate *in vivo* root resorption	Autophagy maintains mineralization under pressure via periostin/β-catenin	Yang et al., 2023

### 3.1 Orthodontic force promotes cementoblast programmed cell death

Basic research has clarified how cementoblasts regulate root resorption under orthodontic force. During mechanical force application, cementoblasts are directly exposed to compressive stress, and one of their earliest responses involves programmed cell death. Both *in vitro* and *in vivo* studies have shown that compressive force induces apoptosis in cementoblasts. Primary human cementoblasts initiate apoptosis 6 h after exposure to pressure (30 g/cm^2^). By 10 h, the proportion of apoptotic cells reaches 18%–20%, accompanied by a significant increase in the levels of pro-apoptotic molecule AXIN1 upregulated 1 (AXUD1) (Diercke et al., 2014). RNA sequencing (RNA-seq) of mouse cementoblasts after pressure application (1.5 g/cm^2^, 8 h) also indicates that the expression of hypoxia- and apoptosis-related genes is upregulated (Liu et al., 2019). In a rat orthodontic model, orthodontic force increases the number of caspase-3- and caspase-8-positive cells on the cementum surface, implying that the apoptotic pathway is activated (Minato et al., 2018). Under pressure conditions, cementoblasts are also direct targets of the adipokine leptin, which upregulates cyclooxygenase-2 (COX-2) expression and promotes PGE2 production by activating ERK1/2 phosphorylation, thereby inducing apoptosis through cytosolic phospholipase A2 (cPLA2) activation and cytochrome *c* release (Ruiz-Heiland et al., 2021). Additionally, compressive force activates ferroptosis via the cGAS-STING/GPX4 axis and consequently suppresses cementoblast mineralization (Wei et al., 2026). This loss of cementoblasts directly disrupts the cellular barrier on the root surface, exposing the underlying mineralized cementum to osteoclasts/odontoclasts and thereby accelerating resorption.

### 3.2 Orthodontic force inhibits cementoblast autophagy

Orthodontic force also inhibits autophagic activity in cementoblasts, compromising their protective function and reducing their mineralization capacity. Autophagy is a highly conserved, lysosome-mediated catabolic process that degrades damaged organelles, protein aggregates, and pathogens, yielding recycled cellular components that contribute to the maintenance of metabolic homeostasis under stress (Liu S. et al., 2023). Autophagy is essential for reversing cementoblast mineralization impairment caused by mechanical stress *in vitro* and *in vivo* (Yang et al., 2023). When severe continuous static pressure is applied to cementoblasts, cell-surface expression of the mechanosensory receptor sphingosine-1-phosphate receptor 1 (S1PR1) markedly decreases. This downregulation suppresses the classical PINK1/Parkin-mediated mitophagy pathway, inhibiting the transcription of RUNX2 and OSX and ultimately impairing cementoblast mineralization; these findings were confirmed *in vitro* and *in vivo* (Wang et al., 2025). Additionally, compressive force markedly upregulates the expression of the long intergenic noncoding RNA lincRNA-p21 in cementoblasts. This lncRNA directly binds forkhead box O3 (FOXO3), resulting in the repression of autophagy-related gene expression, thereby inhibiting autophagy and suppressing mineralized nodule formation. Notably, the knockdown of lincRNA-p21 in a mouse OTM model effectively restored cementoblast mineralization (Liu H. et al., 2022).

### 3.3 Interaction between the inflammatory microenvironment and cementoblasts

Orthodontic force not only directly alters cementoblast function but also induces an aseptic inflammatory response in periodontal tissues, where locally accumulated immune cells and pro-inflammatory mediators create a resorption-promoting microenvironment. On the pressure side, orthodontic loading leads predominantly to the accumulation of M1-polarized (pro-inflammatory) macrophages, which secrete high concentrations of cytokines such as TNF-α and IL-1β (He et al., 2015). These cytokines impair cementoblast function through distinct signaling pathways. IL-1β, a key proinflammatory mediator whose concentrations rapidly increase on the compression side, upregulates miR-325-3p expression, leading, in turn, to the suppression of the expression of the osteogenic transcription factor RUNX2, thus impairing cementoblast differentiation (Wang Y. et al., 2020). Additionally, IL-1β stimulation reduces cementoblast mineralization activity—characterized by decreased CEMP-1 expression and increased BSP expression—an effect exacerbated under compressive force (Diercke et al., 2012b). High-intensity orthodontic loading can upregulate IL-33 expression in periodontal tissues. IL-33 inhibits cementoblast differentiation and mineralization through its receptor ST2 and downregulates the expression of differentiation-related proteins such as RUNX2, OSX, BSP, and OPN (Dong et al., 2020). Similarly, TNF-α upregulates miR-155-3p, which partially mediates the inhibitory effect of TNF-α on cementoblast differentiation. This microRNA represses its target gene potassium channel tetramerization domain-containing 1 (*KCTD1*)—encoding an endogenous inhibitor of canonical Wnt/β-catenin signaling—thereby activating this pathway and suppressing differentiation (Wang et al., 2017). Under inflammatory conditions, TNF-α also exerts its inhibitory effect in part through the fat mass and obesity-associated protein (FTO)/RUNX2 axis (Sun et al., 2022). Chemerin is a recently identified chemotactic protein whose expression is elevated in inflammatory diseases, and Ye et al. (2021) demonstrated that orthodontic force upregulates chemerin expression in periodontal tissues. Chemerin activates the ERK1/2, p38 mitogen-activated protein kinase (MAPK), and PI3K/AKT signaling pathways in cementoblasts through its receptor, ChemR23, thereby inhibiting their differentiation and mineralization. Collectively, these mechanisms cause a loss of cementoblast repair capacity, increasing the severity of root resorption (Ye et al., 2021).

Additionally, the aseptic inflammatory microenvironment increases RANKL secretion by cementoblasts, altering the balance of local bone metabolism. Pro-inflammatory factors such as IL-1β and IL-33 upregulate RANKL expression in cementoblasts, while osteoprotegerin (OPG) expression remains largely unchanged, leading to an elevated RANKL/OPG ratio (Diercke et al., 2012a; Huynh et al., 2017; Dong et al., 2020). Concurrently, chemerin stimulates the release of inflammatory factors, including TNF-α and IL-6, by cementoblasts in a concentration-dependent manner (Ye et al., 2021), leading to osteoclast activation and enhanced root resorption (Nakai et al., 2023). Furthermore, continuous compression induces irreversible senescence in cementoblasts. Senescent cementoblasts are a primary source of local pathological RANKL, which drives the aggregation of apical osteoclasts and mediates pathological root resorption (Zhou et al., 2023). Thus, during rapid tooth movement, cementoblasts play an important regulatory role in resorption by secreting RANKL, enhancing local inflammatory responses, and recruiting additional osteoclasts.

### 3.4 The potential reparative effect of cementoblasts in OIIRR

When orthodontic tooth movement enters the post-lag phase, hyalinized necrotic tissue is completely cleared, the rate of tooth movement increases again, and cementum repair is activated. In the late phase of orthodontic treatment, mechanical stress promotes M2 macrophage polarization through H3 histone acetylation (Wang Y. et al., 2023). Additionally, the intrinsic mechanical response of cementoblasts to mechanical stress contributes to the remodeling of the local immune microenvironment. Under mechanical pressure, cementoblast-derived extracellular vesicles promote macrophage polarization toward the anti-inflammatory M2 phenotype, thereby increasing the proportion of M2 macrophages in the microenvironment and markedly enhancing the efferocytic clearance of apoptotic cells (Yang et al., 2024).

Mechanistic studies have confirmed that macrophage polarization directly affects cementoblast function via exosomes. M2 macrophages secrete anti-inflammatory cytokines, such as IL-10 and transforming growth factor-beta (TGF-β), and deliver microRNAs, including let-7f-5p, via exosomes, resulting in the activation of peroxisome proliferator-activated receptor gamma coactivator 1-alpha (PGC-1α)-dependent mitochondrial biogenesis in cementoblasts. This process upregulates the expression of the mineralization-related proteins BSP, OCN, and OSX, which promotes cementum repair (Zhao et al., 2022; Huang et al., 2024). Concomitantly, under pressure-induced hypoxic conditions, the expression of programmed death-ligand 1 (PD-L1) in cementoblasts is upregulated through a hypoxia-inducible factor 1-alpha (HIF-1α)-driven mechanism. This pathway limits excessive inflammation within the periodontal ligament, thereby modulating the progression of OIIRR (Yong et al., 2022b).

During this stage, orthodontic forces continue to be transmitted through periodontal ligament fibers to the tension-side alveolar bone and root surface (von Bohl et al., 2004). Tensile force promotes cementoblast function through multiple mechanisms. Specifically, tensile force was shown to activate the PTH1R signaling pathway in OCCM-30 cells, causing a time-dependent increase in the mRNA and protein expression of cementoblast markers (RUNX2, OSX, COL1, and OPN) (Li et al., 2018). Additionally, Zhao et al. (2024) demonstrated that tensile force enhances mineralization via the autophagy–periostin–mineralization gene axis. Autophagy mediates mineralization via the periostin/β-catenin axis, and its activation significantly reverses compressive force-induced mineralization inhibition (Yang et al., 2023).

Beyond direct mechanical stimulation, neighboring cell populations in the periodontal microenvironment also contribute to this response. Exosomes derived from human periodontal ligament stem cells promote the proliferation, migration, and mineralization of cementoblasts through the PI3K/AKT signaling pathway (Li et al., 2024). *In vitro* co-culture experiments have also shown that PDLCs and dental pulp stem cells significantly enhance cementoblast proliferation and mineralization capacity (Li Wenwen et al., 2025). Furthermore, cementoblasts induce the differentiation of PDLCs into cementoblasts through paracrine signaling (Sawada et al., 2024). Together, these findings indicate that various cell types in the periodontal microenvironment collectively regulate cementoblast function, thereby influencing cementum repair following OIIRR.

## 4 Protection and repair strategies targeting cementoblasts

Given the critical role of cementoblasts in producing mineralized cementum, a growing number of studies in recent years on OIIRR have focused on enhancing their activity and mineralization capacity to mitigate root damage. Strategies targeting these cells are essential for active tissue repair and improved patient prognosis (Figure 3).

**FIGURE 3:**
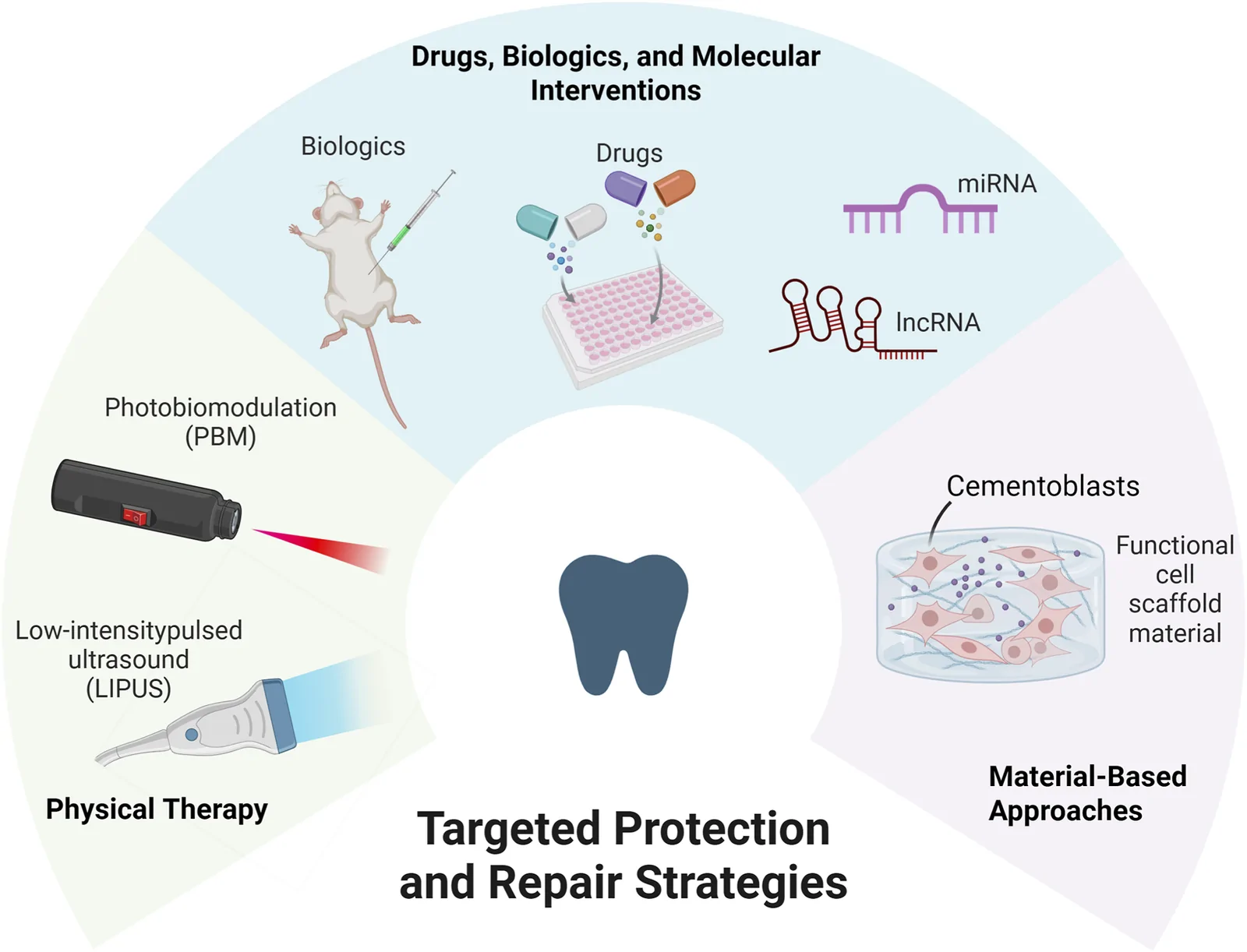
Summary of current protective and reparative intervention strategies targeting cementoblasts in orthodontically induced inflammatory root resorption (OIIRR), encompassing physical therapy, drugs, biologics, molecular interventions, and material-based approaches. Created in BioRender. yue, F. (2026) https://BioRender.com/rxh29bt

### 4.1 Physical therapy

LIPUS is a non-invasive physical therapy applied locally to orthodontically treated teeth, offering the advantage of avoiding systemic side effects. LIPUS inhibits osteoclastogenesis by modulating the OPG/RANKL ratio, thereby reducing odontoclast and osteoclast numbers without affecting tooth movement and significantly decreasing root resorption (Inubushi et al., 2013). *In vitro*, ultrasound stimulation—particularly at 150 mW/cm^2^—promotes cementoblast proliferation, collagen synthesis, and ALP activity while increasing COL1 and ALP expression and upregulating OPG synthesis. These findings indicate that ultrasound stimulation can prevent root resorption and accelerate tissue repair (Dalla-Bona et al., 2008). Zheng et al. (2025) identified a mechanism by which LIPUS alleviates OIIRR. LIPUS remodels the cytoskeleton and upregulates the expression of the nuclear lamina protein lamin A/C, thereby activating the nuclear translocation of the mechanosensitive transcriptional coactivator Yes-associated protein 1 (YAP1). This signaling cascade effectively reverses compressive stress-induced inhibition of cementoblast mineralization, significantly reducing root resorption volume and osteoclast counts in a rat model. Mechanically targeting YAP1 activation provides a novel strategy for non-invasive physical therapy for root resorption (Zheng et al., 2025).

Photobiomodulation (PBM) exerts positive biological effects during OIIRR repair, and its capacity to promote cementoblast mineralization depends strongly on energy density and physical parameters. PBM promotes cementoblast proliferation, migration, and mineralization while inhibiting orthodontic force-induced inflammatory responses, thereby providing a favorable microenvironment for root repair and regeneration. *In vitro* studies have shown that red and near-infrared light PBM, applied with appropriate parameters, significantly enhances early differentiation and mineralization potential in cementoblasts. Irradiating cementoblasts with a 660 nm LED (energy density: 3.2 J/cm^2^) significantly promotes cell migration and upregulates osteogenic and cementogenic gene expression (de Farias et al., 2023). Similarly, irradiating human cementoblasts with a 910-nm gallium-aluminum-arsenide (GaAlAs) semiconductor laser followed by 24 h of culture significantly increases the gene expression levels of *ALP*, *RUNX2*, and *COL1*. This treatment also elevates extracellular ALP activity and the calcium ion concentration, thus enhancing calcified deposit formation following mineralization induction (Nakatani et al., 2024).

Furthermore, the biological effects of PBM are greatly dependent on the precise regulation of physical parameters. A low-level Er: YAG laser enhances cellular repair capacity primarily by activating the MAPK signaling pathway. Fine-tuning the pulse duration alters these cellular responses. Specifically, a longer pulse width (1000 μs) predominantly promotes cell proliferation and migration, whereas a shorter pulse width (100 μs) upregulates expression of mineralization-related genes, both of which ultimately promote cementum formation (Yong et al., 2022c).

A different *in vitro* experiment conducted under tensile stress confirmed that PBM (10.6 µm, continuous mode) increases cell viability over time and enhances cell differentiation after 7 days, while modulating the levels of pro-inflammatory markers such as IL-6, inducible nitric oxide synthase (iNOS), and COX-2. The authors suggested that this supports root repair and reduces inflammatory resorption during orthodontic treatment (Kao et al., 2024). To better mimic the *in vivo* environment, cells were seeded onto root slices from healthy human third molars and irradiated with a diode laser at specific parameters (0.3 W, continuous-wave, 18 J/cm^2^, 60 s/cm^2^). Following irradiation, cells adopted a more cuboidal morphology, the expression of mineralization-related genes was upregulated, mineralized nodules formed, and cementoblast function was enhanced (Bozkurt et al., 2017). *In vivo* animal experiments further demonstrated the protective effect of PBM on tooth roots during orthodontic treatment. In a rat model, diode laser irradiation (650 nm, 30 mW, 5–10 J/cm^2^) protected roots by promoting PDLC cementoblastic differentiation (Kong et al., 2025).

In conclusion, LIPUS and PBM at appropriate parameters promote cementoblast migration and differentiation, accelerating orthodontic tooth movement while reducing root resorption. These physical therapy modalities are therefore promising adjunctive methods for alleviating root resorption during orthodontic treatment. A summary of representative physical therapy modalities (ultrasound and laser) applied to cementoblasts, including their classification, primary biological effects, and underlying molecular mechanisms, is provided in Table 2.

**TABLE 2:** Physical interventions targeting cementoblasts.

Physiotherapy	Experimental model	Type	Mechanisms	References
Ultrasound	*In vitro* (OCCM-30)	Low- and high-intensity pulsed ultrasound (LIPUS/HIPUS, frequency = 1 MHz; SATA intensities = 30 or 150 mW/cm^2^)	Upregulates COL1 and ALP expression; high-intensity US enhances collagen synthesis and ALP activity	Dalla-Bona et al., 2008
	*In vivo* (rat)	Low-intensity pulsed ultrasound (LIPUS, frequency = 1 MHz; spatial-average intensity = 150 mW/cm^2^)	Increases the OPG/RANKL ratio and reduces orthodontically induced root resorption	Inubushi et al., 2013
	*In vivo* (OCCM-30) and *in vitro* (rat)	Low-intensity pulsed ultrasound (LIPUS, frequency = 1.5 MHz; intensity = 30 mW/cm^2^)	Upregulates mineralization-related genes (*Runx2*, *Sp7*, *Bglap*) and protein markers (ALP, RUNX2, OCN); activates YAP1 signaling via the cytoskeleton-lamin A/C pathway	Zheng et al., 2025
Laser	*In vivo* (rat)	Low-level Er: YAG laser (2940 nm; energy densities = 1 or 2 J/cm^2^; pulse duration = 100 or 1000 µs)	Upregulates Lumican and Fibromodulin expression; promotes mineralized nodule formation	Yong et al., 2022c
	*In vivo* (rat)	Photobiomodulation (PBM, 650-nm diode laser, 30 mW, energy densities = 3.3–10 J/cm^2^ [optimally 6.7 J/cm^2^])	Upregulates CAP expression in PDLCs, promoting cementoblast differentiation	Kong et al., 2025
	*In vitro* (OCCM-30)	High-frequency low-intensity semiconductor laser (910 nm, average power = 0.3 W, pulse duration = 200 ns, energy density = 2.0 J/cm^2^)	Increases ALP activity, calcium deposition, and calcified nodule formation	Nakatani et al., 2024
	*In vitro* (OCCM-30)	Photobiomodulation therapy (PBMT, CO_2_ laser 10.6 μm, power = 1.0 W; energy densities = 3, 5, and 10 J/cm^2^)	Increases ALP activity in the late phase, promoting differentiation while modulating inflammation	Kao et al., 2024
	*In vitro* (OCCM-30)	Diode laser biostimulation (940 nm, power = 0.3 W, energy density = 18 J/cm^2^, irradiation time = 60 s/cm^2^)	Increases the mRNA expression of *Ibsp*, *Bglap*, and *Bmp2*/*3*/*6*/7; induces mineralized nodule formation in cementoblasts	Bozkurt et al., 2017

### 4.2 Drugs, biologics, and molecular interventions

Various drugs, biological agents, and small-molecule compounds hold promise for protecting cementoblasts, enhancing their differentiation and mineralization, and regulating the local inflammatory microenvironment. These interventions—categorized as antiapoptotic, prodifferentiation, and anti-inflammatory/antiresorptive actions—alongside their core targets and therapeutic outcomes, are summarized in Table 3.

**TABLE 3:** Drugs, biologics, and molecular interventions for cementoblast protection/regeneration, categorized by mechanism with key targets and outcomes.

Dimension	Representative factor/compound	Core pathway/target	Main biological effects	References
Anti-apoptosis and stress protection	miR-223-3p/*FOXO3*	FOXO3/ROS/mitochondria	Targets FOXO3, thereby reducing ROS levels, restoring mitochondrial function, and mitigating hypoxia-induced apoptosis	He et al., 2025
	L-Arginine/resveratrol	*Sirt1*/autophagy/caspases	Sirt1 upregulation boosts autophagy, inhibiting hypoxia-induced apoptosis and reducing root resorption	Xu et al., 2022
	Adipokines (resistin, visfatin, ghrelin)	HIF-1α/ERK1/2-caspases	Stabilize HIF-1α via ERK1/2, reducing caspase contents and suppressing apoptosis/necrosis under hypoxia	Yong et al., 2021
	Ciliary neurotrophic factor	STAT3/ERK1/2/P38/JNK/autophagy	Induces autophagy but impairs mineralization; blocking STAT3 or ERK1/2 reverses this impairment	Yong et al., 2022a
Promote cementoblast differentiation and mineralization	FGF2/dexamethasone	Wnt signaling pathway (Wnt3a, β-catenin, p-GSK-3β)	Combination synergistically increases ALP activity, nodule formation, and differentiation marker expression	Xie and Shen, 2018
	Lithium chloride (LiCl)	Wnt/β-catenin signaling pathway	Alleviates the mechanical strain-induced suppression of cementoblast differentiation (RUNX2, ALP, COL1)	Ge et al., 2019
	Long noncoding RNA H19 (lncRNA H19)	H19 upregulation	H19 promotes cementoblast differentiation and mineral nodule formation	Hao et al., 2022
	*BMAL1* (circadian clock gene)	Wnt/β-catenin signaling pathway	*BMAL1* promotes cementoblast differentiation and cementum mineralization	Liu S. et al., 2022
	Clock genes (REV-ERBα/SR8278)	Circadian clock network	SR8278-induced inactivation of REV-ERBα promotes cementoblast mineralization	Liu S. et al., 2023
	PTX3	HA/ITGB1/FAK/YAP1 signaling pathway	PTX3 overexpression promotes cementum formation and cementoblast differentiation	(Wang et al., 2024)
	Resveratrol	Inhibition of ERK and AKT phosphorylation/SIRT1	Modulates inflammatory responses (inhibits ERK/AKT pathways), potentially protecting cells and influencing the differentiation status	Negm et al., 2025
	LITTIP-Lgr6-HnRNPK/iPTH	Wnt/β-catenin	LITTIP inhibits cementogenesis; iPTH promotes it by blocking Wnt signaling	Li et al., 2023
	Intermittent parathyroid hormone	PTHR1	Boosts mineralization, ALP, and BSP levels, and nodule formation	Li et al., 2017
	Estrogen (E2)	ERK1/2-OPG-RANKL	Enhances cementogenesis and root repair after orthodontic tooth movement-related resorption	Li et al., 2020
	Irisin	p38 MAPK	Promotes mineralized nodule formation and the expression of differentiation genes	Zhu et al., 2020
​	iPTH/IGF-1	IGF-1	Enhances mineralization via IGF-1 (upregulates CAP, ALP, and OCN)	Li Y. et al., 2021
	Intermittent parathyroid hormone	Periodontal repair	Upregulates BSP, OCN, COL1, and ALP expression and enhances mineralization	Cheng et al., 2023
	Recombinant human growth hormone	ERK1/2	Promotes differentiation and BSP/ALP expression	Wu et al., 2022
	Abaloparatide/PTH (1–34)/PTHrP	PTH1R	Promotes mineralized nodule formation and COL1 expression	Hsu et al., 2023
	17β-Estradiol (E2)	Notch	Promotes late-phase BSP/RUNX2 expression and calcium deposition	Liao et al., 2016
Anti-inflammatory and anti-resorptive	Baicalin	Wnt/β-catenin	Increases OPG and decreases RANKL expression; prevents osteoclasts from attacking the roots	Kunimatsu et al., 2022
	PPARγ agonist (rosiglitazone)	Wnt/β-catenin	Suppresses IL-1β and boosts mineralization (ALP, RUNX2, OCN) by blocking Wnt	Hu et al., 2020
	Senolytics (dasatinib + quercetin)	Clearance of senescent cells	Clears senescent cells, reducing odontoclasts and root resorption	Zhou et al., 2023
	Xanthohumol (XN)	ERK/AKT	Downregulates stress-induced IL-6/COX2 expression and restores ERK/AKT, thereby exerting selective anti-inflammatory effects	Niederau et al., 2022
	Resolvin D1 and resolvin E1	RvD1/E1 receptors/cytokines	Promote mineralization; RvE1 strongly increases OPG, lowers MMPs, and boosts IL-10	Bozkurt et al., 2023
	Circular RNA HIPK3 (circHIPK3)	miR-10b-5p/DOHH/NF-κB	Sponges miR-10b-5p to boost differentiation, resisting TNF-α effects	Zhang et al., 2024
	Transforming growth factor-β1 (TGF-β1)	Smads/AP-1	Upregulates OPG via Smads/AP-1, inhibiting osteoclasts and protecting roots	Mo et al., 2024
	sST2 (IL-33 decoy)/SOD3	SOD3-ERK/IL-33-SOD3	Boost SOD3 mineralization by blocking IL-33; accelerates orthodontic tooth movement and protects roots	Dong et al., 2026

#### 4.2.1 Protection against apoptosis and stress

L-Arginine protects cementoblasts from hypoxia-induced apoptosis by upregulating sirtuin 1 (SIRT1) expression and activating autophagy; *in vivo* studies have also confirmed that L-arginine administration significantly reduces root resorption volume in rats subjected to orthodontic force (Xu et al., 2022). Under hypoxic conditions, adipokines (resistin, visfatin, and ghrelin) inhibit caspase-mediated apoptosis and promote cementoblast survival by activating ERK1/2 and stabilizing HIF-1α expression (Yong et al., 2021). Similarly, miR-223-3p inhibits cementoblast apoptosis under force-induced hypoxia by targeting *FOXO3*, thereby attenuating oxidative stress and mitochondrial dysfunction (He et al., 2025). Ciliary neurotrophic factor (CNTF) functions as a survival factor in various types of peripheral and central neurons, glial cells, and non-neuronal cells. CNTF receptors are expressed on cementoblasts; transient CNTF stimulation activates ERK1/2 and downstream signaling via this receptor complex, thereby protecting cementoblasts from apoptosis and representing a potential target for the prevention and treatment of OIIRR (Yong et al., 2022a).

#### 4.2.2 Promoting cementoblast differentiation and mineralization

Various interventions can restore or enhance cementoblast function through specific signaling pathways in response to orthodontic force-induced inhibition of cementoblast mineralization. Lithium chloride, which activates the Wnt signaling pathway, mitigates the mechanical strain-induced suppression of cementoblast differentiation-related genes such as *RUNX2* and *COL1*. This finding suggests that activation of the Wnt pathway may serve as a viable biomechanical strategy for counteracting mechanical stress-induced damage (Ge et al., 2019). Resveratrol, a polyphenolic compound found in grapes and *Polygonum cuspidatum*, possesses anti-inflammatory, antioxidant, and pro-regenerative properties. It inhibited pressure-induced phosphorylation of the ERK and AKT pathways in cementoblasts, while promoting cell viability in a dose- and time-dependent manner (Negm et al., 2025). When overexpressed, pentraxin 3, a multifunctional glycoprotein involved in extracellular matrix remodeling and the regulation of bone metabolism, promotes cementoblast differentiation and cementum formation via the hyaluronic acid/integrin β1/focal adhesion kinase/YAP1 signaling pathway (Wang et al., 2024). The combined application of fibroblast growth factor-2 (FGF-2) and dexamethasone exerts a synergistic effect via the Wnt signaling pathway, significantly enhancing cementoblast proliferation, ALP activity, mineralized nodule formation, and the expression of differentiation markers (Xie and Shen, 2018). In the context of endogenous and epigenetic regulation, the knockdown of brain and muscle aryl hydrocarbon receptor nuclear translocator-like protein 1 (BMAL1) reduces the levels of the mineralization markers ALP, OPN, and OCN, while decreasing β-catenin expression (Liu S. et al., 2022). REV-ERBs participate in cementum formation and negatively regulate cementoblast mineralization by inhibiting OSX expression, whereas REV-ERBα antagonists effectively activate cementoblast mineralization (Fu et al., 2022; Liu G. et al., 2023), suggesting that the clock gene regulatory axis may also represent a potential intervention target for cementum regeneration. The long noncoding RNA H19 is markedly upregulated during OCCM-30 cell differentiation, and its overexpression upregulates the expression of osteogenic/cementogenic differentiation marker genes and enhances ALP activity and mineral nodule formation without affecting cell proliferation (Hao et al., 2022).

Meanwhile, various systemic hormones also play a role in cementoblast protection and regulation. Among them, PTH and its related protein (PTHrP) analogs are the most thoroughly studied cementoblast protective factors to date. Studies have shown that intermittent PTH (iPTH) not only effectively counteracts the negative effect of mechanical strain on cementoblast differentiation *in vitro*—significantly upregulating the expression of cementoblast markers such as BSP, OCN, COL1, and OSX, and increasing ALP activity and mineralized nodule formation (Li et al., 2017)—but also promotes cementoblast-mediated cementum formation, upregulates cementoblastic protein expression, and enhances cell proliferation in a time-dependent manner in a rat orthodontic root resorption model (Cheng et al., 2023). Mechanistically, iPTH promotes proliferation and suppresses apoptosis via the downstream IGF-1 axis (Li et al., 2021c), while accelerating cementum regeneration through inhibition of the Wnt/β-catenin signaling pathway—an inhibition achieved through the downregulation of LITTIP, a lncRNA that activates Wnt signaling and thus acts as a negative regulator of cementogenesis (Li et al., 2021b; Li et al., 2023). Notably, the synthetic PTHrP analog abaloparatide, which acts on the same receptor (PTH1R), shows greater application prospects than traditional PTH (1–34) in promoting root regeneration due to its “delayed” strong upregulation of type I collagen synthesis in cementoblasts (Hsu et al., 2023).

In addition to the PTH axis, recombinant human growth hormone (rhGH), 17*β*-estradiol (E2), and the exercise-induced factor irisin contribute to the regulation of cementum repair through distinct mechanisms. rhGH exhibits a concentration-dependent effect in promoting cementoblast proliferation. While continuously enhancing the expression of BSP and ALP, rhGH exerts a bidirectional regulatory effect on OPN—promoting its expression at low doses and inhibiting it at high doses—thereby modulating cementum formation (Wu et al., 2022). In an ovariectomy (OVX)-induced model of estrogen deficiency and root resorption, exogenous 17*β*-estradiol supplementation influenced cementoblast differentiation in a time-dependent manner through the Notch signaling pathway. Specifically, it transiently inhibited differentiation during the early phase, promoted BSP and RUNX2 expression in the later phase (after 10 days), and enhanced mineralized nodule formation at 21 days (Liao et al., 2016; Li et al., 2020). Furthermore, irisin—a polypeptide hormone secreted primarily by muscle in response to exercise—enhanced the intrinsic mineralization capacity of cementoblasts, aiding in root surface repair and promoting new cementum formation (Zhu et al., 2020). In summary, various pharmacological agents and biological molecules synergistically regulate cementoblast proliferation, differentiation, and mineralization through an intertwined signaling network, providing a theoretical foundation for targeted interventions in cementum regeneration.

#### 4.2.3 Anti-inflammatory and antiresorptive strategies

Regarding anti-inflammatory mechanisms and the inhibition of osteoclastogenesis, the PPARγ agonist rosiglitazone suppresses TNF-α-induced IL-1β expression, reduces the inflammatory response, promotes the expression of ALP, RUNX2, and OCN, and increases mineralized nodule formation by inhibiting the Wnt/β-catenin signaling pathway in cementoblasts. This indicates that PPARγ exerts dual anti-inflammatory and pro-mineralization protective effects (Hu et al., 2020). In an inflammatory microenvironment simulated by treatment with exogenous TNF-α, the expression of the circular RNA circHIPK3 is significantly downregulated. The overexpression of circHIPK3 activates the miR-10b-5p/DOHH/NF-κB signaling axis, which partially reverses the TNF-α-mediated suppression of cementoblast differentiation and mineralization, attenuates inflammation-induced cementum destruction, and promotes cementum regeneration (Zhang et al., 2024). Specialized pro-resolving mediators, such as resolvin D1 (RvD1) and resolvin E1 (RvE1), promote the resolution of inflammation, restore tissue homeostasis, and enhance cementoblast proliferation and mineralization. Compared with RvD1, RvE1 induces a greater upregulation of mineralization-related genes and the anti-osteoclastogenic factor OPG and exerts a stronger inhibitory effect on the expression of RANKL and RANK. RvE1 also significantly upregulates its receptor, ChemR23, as well as the anti-inflammatory cytokine IL-10, illustrating its potential as a promising target for periodontal regeneration (Bozkurt et al., 2023). Xanthohumol, an isoprenylated plant polyphenol found in hops (*Humulus lupulus*), exhibits anti-inflammatory and anti-angiogenic properties. Xanthohumol selectively downregulates compressive force-induced IL-6 inflammatory responses by inhibiting ERK/AKT phosphorylation and enhances cementoblast viability at low concentrations (Niederau et al., 2022).

The natural flavonoid compound baicalin upregulates the expression of OPG and suppresses that of RANKL in human cementoblasts in a concentration-dependent manner. This regulatory effect depends on activation of the Wnt signaling pathway, suggesting that baicalin can modulate the cementum immune microenvironment (Kunimatsu et al., 2022). In addition, oral administration of combined dasatinib and quercetin significantly reduces root resorption during OTM by targeting and clearing mechanically stress-induced senescent RANKL^+^ cells (Zhou et al., 2023). In an *in vivo* orthodontic tooth movement model, the local administration of TGF-β1 promoted OPG expression by inducing the binding of Smad-related proteins and activator protein-1 (AP-1) to the OPG promoter and enhancing its transcription in cementoblasts. The consequent increase in OPG secretion inhibited osteoclast differentiation and protected cementum from excessive resorption (Mo et al., 2024).

Traditional root protection strategies often function by broadly inhibiting osteoclasts, which inadvertently delays alveolar bone remodeling and slows orthodontic tooth movement. Consequently, specifically enhancing the reparative capacity of cementum within a pro-remodeling microenvironment remains a central challenge. Recently, Dong et al. (2026) showed that superoxide dismutase 3 (SOD3) promotes mineralization significantly more effectively in cementoblasts than in preosteoblasts, revealing a cell-type-specific redox regulatory mechanism. Exogenous enhancement of SOD3 signaling protects cementoblasts even in the presence of active osteoclasts, providing a biological rationale for targeting cementoblasts to treat root resorption and suggesting that this therapeutic challenge may be resolved by specifically amplifying cementoblast repair signals (Dong et al., 2026).

### 4.3 Material-based approaches

The interaction between cementoblasts and biomaterial scaffolds is an important determinant of successful cementum regeneration and periodontal tissue reconstruction. Studies have investigated how functional hydrogels and electrospun polymer scaffolds modulate cementoblast behavior and the local microenvironment across multiple functional dimensions and how they influence bone regeneration.

First, functionalized hydrogel carriers offer distinct advantages in modulating the local microenvironment and promoting mineralization. In particular, direct *in vitro* contact between cementoblasts and gellan gum-xanthan gum composite hydrogels loaded with an artificially synthesized amelogenin-derived peptide (ADP-5) was shown to significantly upregulate the expression of vascular endothelial growth factor A (VEGFA) and IL-11, while concurrently downregulating that of the pro-inflammatory mediator TNF-α. This indicates that ADP-5-functionalized hydrogels effectively modulate the local immune and inflammatory microenvironment and thereby promote tissue healing. Furthermore, these hydrogels significantly increase extracellular calcium nodule deposition in cementoblasts, confirming their strong potential to promote cementum-like mineralization (Attik et al., 2023).

Second, electrospun polymer scaffolds designed for cementoblast delivery provide a suitable three-dimensional microenvironment for the growth of these cells. Electrospun polycaprolactone (PCL) scaffolds prepared at 10% and 20% (*w/v*) concentrations were evaluated as delivery vehicles for seeded OCCM-30 cementoblasts. These scaffolds, particularly the 10% PCL formulation, exhibited favorable cytocompatibility and supported cementoblast delivery, significantly enhancing cell differentiation and extracellular matrix mineralization *in vitro*. This indicated that this scaffold system has potential for application in periodontal and cementum regeneration (Abdalla et al., 2022).

Additionally, different bone regeneration materials exert material-specific and time-dependent biological effects on cementoblasts. In an *in vitro* study comparing conditioned media from Bio-Oss®, Endobon®, Nanobone®, and Emdogain® on immortalized mouse cementoblasts (OCCM-30), all tested materials promoted cell migration. Regarding osteogenic differentiation, Bio-Oss® induced the highest expression of BSP, RUNX2, COL1, and ALP on day 3. Conversely, Nanobone® sustained the upregulation of BSP and COL1 through day 6 and produced the highest degree of mineralized nodule formation as evaluated by von Kossa staining. This suggested that different scaffold materials preferentially promote either cell differentiation or mineralized nodule formation (Mutafcilar Velioglu et al., 2025).

In summary, biomaterial-based approaches—including bioactive peptide-functionalized hydrogels, biocompatible electrospun scaffolds, and commercial bone graft substitutes—influence cementoblast responses through distinct mechanisms. These materials modulate the local microenvironment, promote cell migration, and induce cementum matrix mineralization, providing functional design strategies for future periodontal regeneration scaffolds.

## 5 Conclusions and future perspectives

In this review, we comprehensively examined the role of cementoblasts in OIIRR. Cementoblasts act as major regulators of orthodontic root resorption and repair. They directly sense orthodontic forces and adjust the expression of mineralization-related genes, thereby controlling cementum deposition and remodeling. Rather than acting in isolation, cementoblasts function as a central node in the periodontal microenvironment, establishing complex paracrine networks with macrophages, periodontal ligament stem cells, and pre-osteoclasts to translate mechanical stimuli into multilevel molecular signaling cascades. Under excessive orthodontic force, cementoblast apoptosis and premature senescence directly impair intrinsic repair mechanisms. Concurrently, affected cementoblasts promote local inflammation and elevate the RANKL/OPG ratio, thereby driving osteoclast recruitment and exacerbating root resorption. However, this resorptive process is reversible. Relieving orthodontic force or applying targeted physical and pharmacological interventions can reactivate cementoblast function. Therapeutic approaches such as LIPUS and iPTH administration have been shown to effectively activate the secretory function and mineralization capacity of cementoblasts by modulating signaling pathways, including the Wnt/β-catenin pathway. These interventions promote cementum-like matrix deposition within resorptive lacunae, ultimately achieving the partial restoration of structural integrity and functional performance.

Regarding orthodontic clinical translation, the functional homeostasis of cementoblasts influences susceptibility to root resorption and repair potential. Continuous excessive force leads to cell apoptosis and premature senescence, underscoring the importance of optimizing force-application methods through the use of intermittent light forces whenever possible. Furthermore, local auxiliary interventions show evident synergistic value. Although these physical or pharmacological methods are currently mostly at the animal experimental stage, large-scale, well-designed clinical trials will be required to evaluate the safety and efficacy of these therapies. Future research can establish a precise treatment system centered on cementoblasts, integrating multidisciplinary approaches in mechanics, materials science, and regenerative medicine to support mineralization, cementum regeneration, and microenvironmental immune remodeling. This approach aims to transform the current root resorption management mode of “passive monitoring and delayed treatment,” providing patients with safer, more efficient, and individualized clinical decisions while ensuring orthodontic efficacy.

## Glossary

**OIIRR**: Orthodontically induced inflammatory root resorption
**LIPUS**: Low-intensity pulsed ultrasound
**PTH**: Parathyroid hormone
**TNF-α**: Tumor necrosis factor-α
**PGE2**: Prostaglandin E2
**Piezo1**: Piezo-type mechanosensitive ion channel component 1
**TRAP**: Tartrate-resistant acid phosphatase
**RANKL**: Receptor activator of nuclear factor-κB ligand
**RANK**: Receptor activator of nuclear factor-kappa B
**NF-κB**: Nuclear factor kappa-B
**AP-1**: Activator protein-1
**OPN**: Osteopontin
**BSP**: Bone sialoprotein
**RUNX2**: Runt-related transcription factor 2
**OSX**: Osterix
**CEMP-1**: Cementum protein 1
**CAP**: Cementum Attachment Protein
**OCN**: Osteocalcin
**ALP**: Alkaline phosphatase
**COX-2**: Cyclooxygenase-2
**ERK1/2**: Extracellular signal-regulated kinase 1/2
**cGAS-STING**: Cyclic GMP-AMP synthase-stimulator of the interferon genes pathway
**GPX4**: Glutathione peroxidase 4
**S1PR1**: Sphingosine-1-phosphate receptor 1
**PINK1**: PTEN-induced kinase 1
**PARKIN**: Parkin RBR E3 ubiquitin-protein ligase
**Wnt/β-catenin**: Wingless-type MMTV integration site family/beta-catenin
**MAPK**: Mitogen-activated protein kinase
**PI3K**: Phosphatidylinositol-3-kinase
**AKT**: Protein kinase B
**OPG**: Osteoprotegerin
TGF‐β: Transforming growth factor-β1
**HIF-1α**: Hypoxia-inducible factor 1-alpha
**PTH1R**: Parathyroid hormone 1 receptor
**PTHrP**: Parathyroid hormone-related protein
**PPARγ**: Peroxisome proliferator-activated receptor gamma
**Sp7**: Sp7 transcription factor
**Bglap**: Bone gamma-carboxyglutamate protein
**Bmp2**: Bone morphogenetic protein 2
**Ibsp**: Integrin-binding sialoprotein
**FoxO3**: Forkhead box O3
**SIRT1**: Silent mating type information regulation 2 homolog 1
**HIF-1α**: Hypoxia-inducible factor-1 alpha
**STAT3**: Signal transducer and activator of transcription 3
**HA**: Hyaluronic acid
**ITGB1**: Integrin beta-1
**FAK**: Focal adhesion kinase
**IGF-1**: Insulin-like growth factor 1
**SOD3**: Superoxide dismutase 3
**JNK**: c-Jun N-terminal kinase
**ROS**: Reactive oxygen species
**MDA**: Malondialdehyde
**FTO**: Fat mass and obesity-associated protein
